# Texas Escapes Epidemic

**Published:** 1905-11

**Authors:** 


### TEXAS MEDICAL JOURNAL.

AUSTIN, TEXAS.

A MONTHLY JOURNAL OF MEDICINE AND SURGERY.

EDITED AND PUBLISHED BY
F. E. DANIEL, M. D.
Mrs. F. E. DANIEL, Business Manager.

Published Monthly at Austin, Texas. Subscription price $1.00 a year in advance

### TEXAS ESCAPES EPIDEMIC.

Again Texas is to be congratulated upon her exemption from yellow fever, when the sister States, Louisiana, Mississippi, Tennessee, and Florida, and the neighboring Republic of Mexico, have been afflicted, despite their superior facilities for preventing it. I say the superior facilities. I mean that in those States there are well-organized Boards of Health with ample means and authority; while in Texas we have a single officer, whose powers are limited and to whom our wise legislatures have denied even one assistant, and curtailed the appropriation to the extent of seriously crippling him in the discharge of his duties. Nevertheless, with no office assistant except a layman,—the intelligent and efficient young cavalry captain, Walker—who is secretary, stenographer, typewriter, and per necessity, Assistant State Health Officer, he, Dr. Tabor, has successfully guarded the State from invasion when threatened on all sides and throughout her extensive border, and the many gates through which trade and travel enter the State. The credit is due Dr. Tabor; and the more credit by reason of the terrible handicap under which he is placed. The State and the people owe him a lasting debt of gratitude. There should be some public acknowledgment of it, and we are gratified to note that the Chamber of Commerce of both Dallas and Waco have publicly recognized Dr. Tabor's service to the State and propose a public testimonial.

Notwithstanding what we have said above, and the fact that single-handed and crippled Dr. Tabor has kept yellow fever out of the State, we question very seriously if it would not be the part of wisdom and that "economy" so dear to the hearts of our Solons in the Legislature, to turn over the matter of epidemic invasions to the Federal government altogether. Unless the State will deal with the matter in a more liberal manner, and give us a Board of Health whose duties shall be the instituting and enforcement of sanitary measures for the protection of the people from other than quarantinable diseases, and with a liberal appropriation with which to enforce the authority, I believe it would be best to let the Marine Hospital Service look after international and interstate quarantine, leaving our State Health Officer or a Board of Health free to look to the prevention of those diseases which originate in our midst, e. g., typhoid fever, diphtheria, etc., and which are easily preventable; and to measures for the arrest of the spread of consumption. The small yearly appropriation for public health, all of which, and more, is expended on quarantine,* could be made to do much towards the prevention of other diseases. The prevention of pollution of drinking water alone, to say nothing of rigidly enforcing disinfection of cars, hotels, schoolhouses, churches, etc., would do much to diminish the death rate. Texas has had to call on the M. H. S. more than once, notably in 1882 and in 1896; and without its assistance this year, doubtless the epidemic of yellow fever would have been more widespread and fatal throughout the South than it has been. Why not, then, let Uncle Sam take charge of the matter, as he has done with regard to commerce and travel? The maintenance by Texas of an inspection service at three points on the Rio Grande is an absurdity, seeing that the river is fordable anywhere for a thousand miles; and it is a waste of money that could be expended to better purpose in looking after preventable diseases other than yellow fever. Where one person has died of yellow fever in the last hundred years, one hundred and fifty have died of consumption. I yield the question of States rights, which for many years I have defended, seeing that Texas is too poor or too mean to enforce those rights by instituting a broader and more enlightened policy backed by sufficient means to make it effective. She pays her "one-man band" — the zealous and popular and efficient Tabor—just $208 a month. He does not want it any more after the present term.

---

*The annual appropriations for all sanitary purposes in Texas is about $45,000. This year $10,000 deficiency warrants were issued by the Governor and Dr. Tabor virtually borrowed the money on them from Galveston banks. The appropriation of Louisiana is $140,000 a year. The M. H. S. expended a quarter million in New Orleans during the recent epidemic of yellow fever.

Dr. R. H. Harrison, Sr., of Columbus, Texas, died at his home in that city October 17, 1905, aged 78 years. Dr. Harrison was a native of Kentucky and practiced medicine many years at Hickman, in that State. He received a diploma in 1873 from the Alabama Medical College at Mobile, and settled in that year at Columbus, Texas, where he resided continuously to the date of his death. Dr. Harrison was a prominent figure in the medical profession and in State politics for many years, and was one of the best known and most popular medical men in the State. He was president of the Texas State Medical Association in 1876 and served several times as chairman of the Association's Committee of Medical Legislation and chairman of its Section on Public Health and Hygiene. He made many valuable contributions to medical literature, most of which are preserved in the Transactions of the Texas State Medical Association. His paper on the "Medical and Surgical Aspects of the Galveston Flood," was published in the Texas Medical Journal of October, 1901. Dr. Harrison was extensively known as an authority on yellow fever. We will miss the able, popular, enthusiastic and ever genial old doctor at our annual reunions. Peace to his ashes. "Well done thou good and faithful servant, enter upon thy reward."

---

A Literary "Gem" of impurest ray,—serene or otherwise; and with a flaw in it. Our friend, Dr. Fassett, of the *Medical Herald,* publishes an alleged poem entitled, "The Dying Speech of Meiamoun" by Homer Clark Bennett, M. D., M. E. Lima,, Ohio with the following foot notes, and calls it a "literary gem."

Note.—This rare adaptation from Gautier was first written in liquid prose by Dr. C. A. L. Reed, and published in *The Medical Mirror,* in April, 1897. In October, 1898, this was reprinted in the *Mirror,* together with Dr. Bennett's beautiful poetical version printed above. In response to many inquiries for copies of this poem, which is now out of print, it has been deemed worthy of reproduction, as a literary gem.

*Meiamoun, a tiger hunter, was granted the bliss of a single night with Cleopatra, upon condition that he should die on the morrow. He willingly accepted the terms. On taking the poisoned cup at daybreak, he is presumed to pause only to make this speech, the final words of which are interrupted by the arrival of Marc Antony's herald.

Dr. Bennett's "beautiful version" fairly reeks with nastiness. It is the most libidinous, the most obscene thing I ever saw in print. The doctor has a juicy imagination that would have done credit to the Marquis de Sade. He describes minutely the disgusting features of the supposed night's performances, omitting nothing. It is the concentrated impurities of the Songs of Soloman, Michelet's L'Amour, Don Juan, Venus and Adonis, Lola Montez and Camille, boiled down. Tolstoy's "Resurrection" with the realistic seduction-rape chapter was too clean to contribute to the make-up of the description of "A night of bliss with Cleopatra"—(old Cleopatra—a jaded prostitute!). The author should be ashamed of it. Such literature has no place in any decent medical journal or any other journal. Suppose the young daughter of either of these gentlemen, or of any other man, should get hold of it? The most abandoned Cyprian should blush in the dark to hear such a scene so described. I am by no means a prude, but I confess that reading this "gem" made me sick at the stomach. I will not, for sheer decency's sake quote even a line of it, but I want to say that the lascivious language—revolting enough,—is not the author's only sin. No amount of poetic license will justify or excuse a solecism like this; this is the flaw in the "gem" as a literary production: "So did'st I (sic) in more eagre quest pursue thy leading charms." * * * "Then I did'st feast (sic) my vision," etc.

I can not understand what excuse Dr. Fassett found for besmirching the *Herald* with such an unclean and immoral production. "Prithee, an ounce of civet, good Mr. Apothecary, to sweeten my imagination," and take the taste out of my mouth.

